# Optical limiting via spontaneously generated coherence

**DOI:** 10.1038/s41598-023-27381-1

**Published:** 2023-01-07

**Authors:** Abbas Silatan, Mohsen Ghaderi GoranAbad, Mohammad Mahmoudi

**Affiliations:** Department of Physics, University of Zanjan, University Blvd, 45371-38791 Zanjan, Iran

**Keywords:** Nonlinear optics, Nonlinear optics

## Abstract

We investigate the reverse saturable absorption (RSA) and optical limiting (OL) in a three-level V-type quantum system considering the effect of the spontaneously generated coherence (SGC). It is shown that in the absence of the SGC effect, the saturable absorption (SA) is dominant in the system. By taking into account the SGC effect, we prove that the SA dramatically switches to the RSA. Moreover, it is demonstrated that the OL threshold and OL efficiency can be controlled by the SGC effect. In addition, we show that the applied field properties such as detuning can modify the SGC-induced optical limiter efficiency. It is also shown an increase in the atomic density and length of the medium makes the optical limiter more efficient. The analytical calculation shows that the Kerr nonlinearity caused by the SGC effect is the main mechanism of the system evolution. Finally, the theoretical Z-scan experiment is presented to confirm the obtained results. Our proposed scheme can be useful to design controllable atomic optical limiters for optical devices with different sensitivities.

## Introduction

In recent decades, the advancement of technology has increased the need to use laser fields in both laboratory and industrial fields. This need has led to great attention to the development of innovations and ideas for the protection of optical elements against high-intensity lasers. The human eye and other optical and electronic devices and sensors may be damaged when exposed to high intensities of laser light. The development of optical phenomena relying on atomic coherence and quantum interference created by laser fields has led to the emergence of devices that have high controllability and efficiency. Optical limiting (OL)^1,2^ and All-optical switching^3,4^ are among these widely used phenomena that are created by controlling the transmission of light. Optical limiters are tools that can attenuate the transmission of light, and thus their presence in optical configurations is essential. When the intensity of the light entering the optical device exceeds a certain limit, which is defined as the optical threshold, the optical limiter enters into action and does not allow light to be emitted beyond this threshold. A wide range of materials in which optical limitation was inherently observed have always been traditionally used. These nonlinear materials mainly contain organic and inorganic materials^5–7^ such as quantum dots^8^, metal nanoclusters^9,10^ and carbon based materials^11–14^. Although Azzam et al. showed that the OL behavior depends on the density and concentration of the molecules in thin films^15^, generally the main characteristics of an optical limiter, including the OL threshold and OL range of materials that are inherently optical limiters, cannot be controlled. The design of comprehensive optical limiters with controllable features can be used to protect optical devices with different sensitivities. In recent years, Mahmoudi et al. have proposed an atomic optical limiter whose OL behavior characteristics can be controlled by coherent light intensity, as well as magnetic field^16^. In another work, this group presented a simple scheme in which they showed that the OL threshold and OL region in a three-level pump-probe V-type quantum system can be modified by a microwave field^17^.

From a physical point of view, the two main mechanisms governing the generation of OL are based on the reverse saturable absorption (RSA)^18–20^ and nonlinear scattering^21–23^. When the RSA governs in a material, the absorption of the excited states is larger than the absorption of the ground state, leading to an increase in the absorption by increasing the intensity of the applied light. In contrast, absorption properties disappear gradually in the SA materials due to the depletion of the ground state.

On the other hand, atomic coherence created by spontaneous emission is often referred to as vacuum induced coherence or spontaneously generated coherence (SGC) and has been widely studied in recent years. The effect of State superpositions created by spontaneous emission was introduced by Javanainen^24^. SGC is rooted in the interference between two spontaneous emissions of transitions from two close-lying levels to the common lower energy level with non-orthogonal dipoles in the quantum system. However, finding such conditions are difficult in real atomic system in free isotropic space^25^. It was suggested that the SGC can be generated in anisotropic vacuum, even for orthogonal dipole moment^26^. Several methods such as putting the atom inside a multilayer dielectric plate cavity^27^, coupling of upper two levels by applying a microwave field^28,29^, and applying the laser fields have been introduced to simulate the SGC^30^. Ficek and Swain proposed an alternative scheme that makes it possible to observe the effect of SGC on optical phenomena without considering the parallel dipole moments. In this scheme, the ground state is coupled to only one of the excited states with a laser field, and the two upper levels with perpendicular dipole moments are coupled together by applying a dc field. It was shown that the scheme is equivalent to the usual V-type three-level system with parallel dipole moments of transitions^31^.

In general, large intensities are necessary to modify the absorption properties of the medium, however, many nonlinear phenomena can be established in low intensities in the presence of quantum interference. In addition, quantum interference between different decays from upper levels can reduce the relaxation of the atomic coherence due to the spontaneous emission. These are the reasons why the role of SGC has been intensively investigated in various nonlinear phenomena^32^. The effect of the SGC on the absorption spectrum has been experimentally investigated in a V-type^33^, Y-type and N-type atomic systems^34^. Various theoretical works have been done to determine the role of the SGC in the bistability and mulistability^35,36^, atom-photon entanglement^37,38^ in different atomic systems. Recently, it has been shown that SGC is capable of transferring the orbital angular momentum from a Laguerre–Gaussian field to a weak plane wave^39^. Moreover, Mahmoudi et.al showed that atoms can be cooled to even below recoil temperature under a cooling mechanism based on SGC-induced electromagnetically induced transparency^40^.

In this paper, we take advantage of the coherence induced by SGC to introduce a simple controllable optical limiter. We consider a V-type three-level atomic system in the presence of the SGC to investigate the RSA and the corresponding OL behavior. By creating SGC and increasing its value, it is seen that the SA in the atomic system switches to the RSA, leading to an efficient OL. Moreover, it is shown that the OL threshold and OL ability to attenuate the transmission of light can be coherently modified in the presence of SGC. Our analytical solution shows that the physics governing the results is based on the Kerr nonlinearity due to the SGC. Ultimately, the SGC-induced OL is confirmed by the calculation of the theoretical Z-scan technique. Our suggested atomic scheme can be useful for designing optical limiters with controllable features to protect optical devices with different sensitivities.

## Theoretical framework

**Figure 1:**
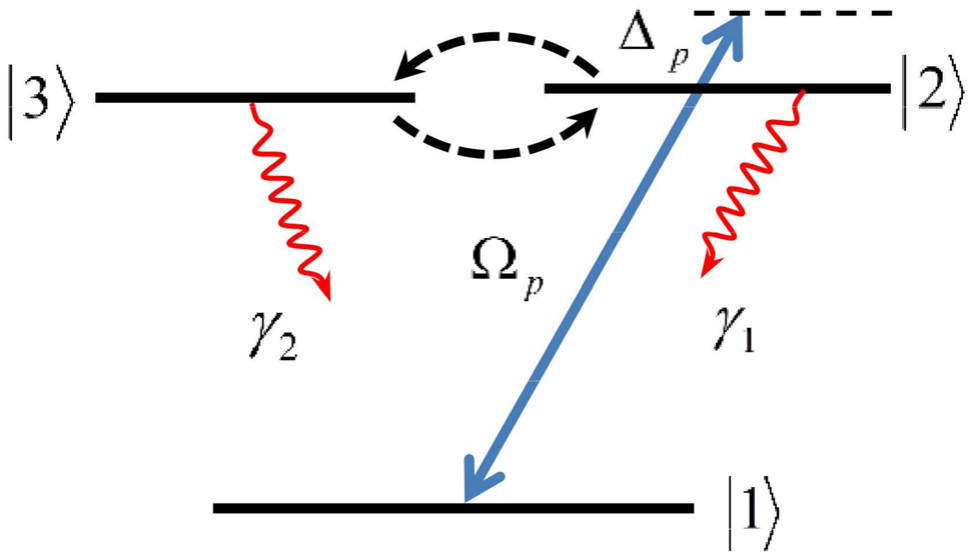
Schematic of a three-level V-type atomic system in the presence of SGC effect.

We introduce a three-level V-type quantum system in the presence of the SGC effect as has been shown in Fig. 1. The lower level and two near generated upper levels are shown by $|1\rangle$, $|2\rangle$ and $|3\rangle$, respectively. The SGC parameter ($\eta =\vec {\mu }_{31} \cdot \vec {\mu }_{21} /\left| \vec {\mu }_{31}\right| \left| \vec {\mu }_{21}\right|$) denotes the alignment of the two dipole moments, which represents the strength of the interference in spontaneous emission. Here, $\vec {\mu }_{31}$ and $\vec {\mu }_{21}$ stand for electric dipole moment of corresponding transitions. Rabi-frequency between transition $|1\rangle \rightarrow |2\rangle$ is specified as $\Omega _{p}=\varepsilon _{p} \cdot \vec {\mu }_{12}/\hbar$, where $\hbar$ is the Planck’s constant. $\varepsilon _{p}$ is considered as the probe field amplitude. Also, the SGC parameter is applied between two levels $|3\rangle$ and $|2\rangle$. The spontaneous decay rates from level $|3\rangle$ to the level $|1\rangle$ and from level $|2\rangle$ to the level $|1\rangle$ is devoted by $\gamma _{2}$ and $\gamma _{1}$, respectively. By applying the dipole and rotating wave approximations, the interaction Hamiltonian for the desired system is considered as follows:

1 $$\begin{aligned} H_{int}=-\hbar \Omega _{p} e^{-i\Delta _{p}t}\left| 2\right\rangle \left\langle 1\right| +H.c. \end{aligned}$$

Here, $\Delta _{p}=\omega _{p}-\omega _{21}$ is defined as the probe field detuning and $\omega _{p}$ is introduced as the frequencies of the probe field. While $\omega _{21}$ is considered as the atomic resonant frequency of the transition $|2\rangle -|1\rangle$. By solving the steady-state of the density matrix equations (Eq. 2) and the calculation of the probe transition coherence $\rho _{21}$, we can explain the response of the medium to the probe field.

2 $$\begin{aligned} {\dot{\rho }}_{22}= & {} -2\gamma _{1}\rho _{22}+i\Omega _{p}\rho _{12} -i\Omega _{p}\rho _{21}-\eta \sqrt{\gamma _{2}\gamma _{1}}(\rho _{32}+\rho _{23}),\nonumber \\ {\dot{\rho }}_{33}= & {} -2\gamma _{2}\rho _{33} -\eta \sqrt{\gamma _{2}\gamma _{1}}(\rho _{32}+\rho _{23}),\nonumber \\ {\dot{\rho }}_{12}= & {} (-i\Delta _{p}-\gamma _{1})\rho _{12} +i\Omega _{p}(\rho _{22}-\rho _{11})-\eta \sqrt{\gamma _{2}\gamma _{1}}\rho _{13},\nonumber \\ {\dot{\rho }}_{13}= & {} -\gamma _{2}\rho _{13}+i\Omega _{p}\rho _{23} -\eta \sqrt{\gamma _{2}\gamma _{1}}\rho _{12},\nonumber \\ {\dot{\rho }}_{23}= & {} [i\Delta _{p}-(\gamma _{2}+\gamma _{1})]\rho _{23} +i\Omega _{p}\rho _{13}-\eta \sqrt{\gamma _{2}\gamma _{1}}(\rho _{22}+\rho _{33}),\nonumber \\ {\dot{\rho }}_{11}= & {} -({\dot{\rho }}_{22}+{\dot{\rho }}_{33}). \end{aligned}$$

We are going to introduce the governing equations in theoretical issues of optical limiting. In an atomic medium, the polarization vector is given by

3 $$\begin{aligned} \vec {P}(z, t)=\chi _{p} \vec {\varepsilon _{p}} e^{-i\left( \omega _{p} t-k_{p} z\right) }+c.c., \end{aligned}$$

where $k_{p}$ is the probe wave vector. The response of the medium to the probe field is determined by susceptibility $\chi _{p}$.

4 $$\begin{aligned} \nabla ^{2} \vec {E}_{p}-\mu _{0} \varepsilon _{0} \frac{\partial ^{2} \vec {E}_{p}}{\partial t^{2}}-\mu _{0} \frac{\partial ^{2} \vec {P}}{\partial t^{2}}=0. \end{aligned}$$

$\vec {E}_{p}$ is a weak linearly probe field that is determined as $\vec {E_{p}}(z,t)=\vec {\varepsilon _{p}}(z)e^{-i\left( \omega _{p}t-k_{p}z\right) }$. $\varepsilon _{0}$ and $\mu _{0}$ are the permittivity and permeability of vacuum, respectively. By solving the wave equation Eq. (4) and using slowly varying approximation, a simplified model of the equation is specified as Eq. (5) that helps us to obtain the output probe field amplitude:

5 $$\begin{aligned} \frac{\partial \varepsilon _{p}}{\partial z}=\, & {} i 2 \pi \omega _{p}\left( \mu _{0} \varepsilon _{0}\right) ^{1 / 2} \varepsilon _{p} \chi _{p}, \end{aligned}$$

6 $$\begin{aligned} \varepsilon _{p}(z=l)=\, & {} \varepsilon _{p}(0) e^{i 2 \pi k_{p} l x_{p}}, \end{aligned}$$

7 $$\begin{aligned} \chi _{p}=\, & {} \frac{n \mu _{21}^{2} \rho _{21}}{\hbar \Omega _{p}}, \end{aligned}$$

with *l* being as the thickness of the sample. As shown in Eq. (7), $\chi _{p}$ is proportional to *n* (density of atoms) and $\rho _{21}$ which can be calculated from Eq. (2). By changing the parameters and simplification, $\varepsilon _{p}(z=l)$ is given by

8 $$\begin{aligned} \varepsilon _{p}(z=l)=\varepsilon _{p}(0) e^{i \frac{\alpha l \rho _{21} \gamma }{2 \Omega _{p}}}, \end{aligned}$$

where $\alpha l=\frac{4 \pi n \mu _{21}^{2} k_{p} l}{\hbar \gamma }$ is the resonant absorption and $\gamma _1=\gamma _2=\gamma$. By normalizing susceptibility as $S_{p}=\frac{\gamma \rho _{21}}{\Omega _{p}}$, the output probe field amplitude is determined as Eq. (9):

9 $$\begin{aligned} \varepsilon _{p}(z=l)=\varepsilon _{p}(0) e^{i \frac{\alpha l}{2} S_{p}}. \end{aligned}$$

The normalized transmission of the probe field is considered as the ratio of output intensity to input intensity. It can be related to $S_{p}$, a complex quantity whose imaginary part stands for the absorption of the probe field.

10 $$\begin{aligned} T=\frac{\left| \varepsilon _{p}(z=l)\right| ^{2}}{\left| \varepsilon _{p}(0)\right| ^{2}}=e^{-\alpha l {\text {lm}}\left[ s_{p}\right] }. \end{aligned}$$

## Results and discussion

**Figure 2:**
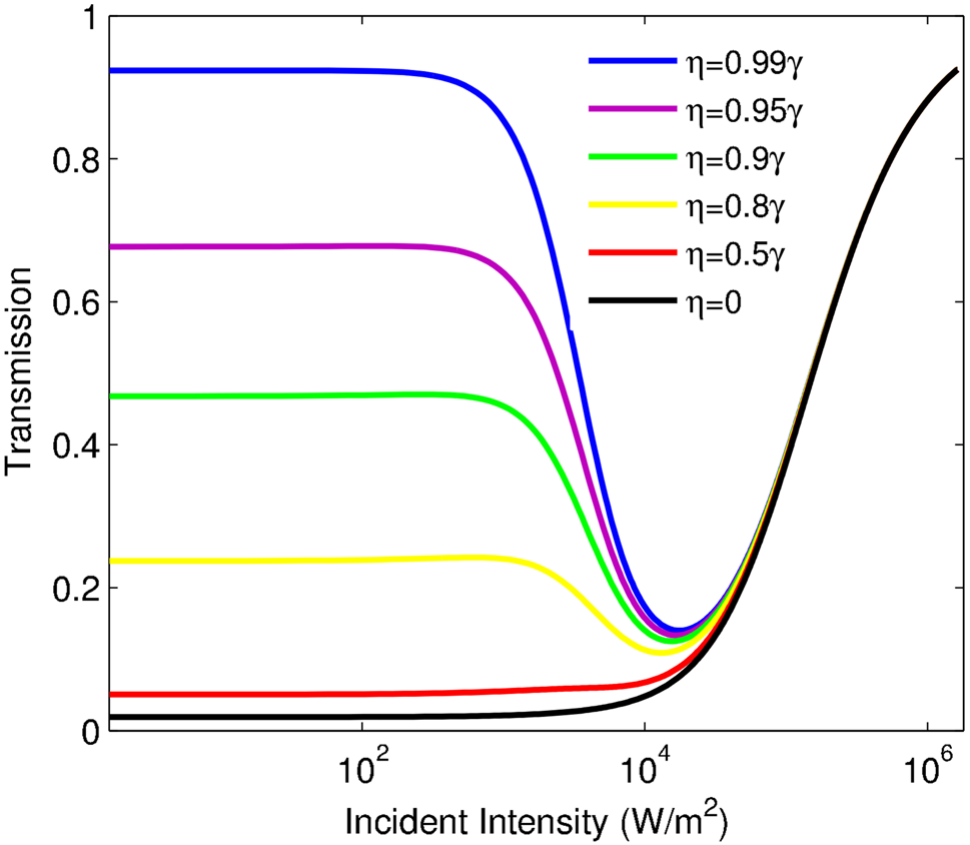
Transmission of the probe field versus incident intensity for different values of $\eta$: $\eta =0.99\gamma$ (blue line), $\eta =0.95\gamma$ (purple line), $\eta =0.90\gamma$ (green line), $\eta =0.8\gamma$ (yellow line), $\eta =0.5\gamma$ (red line) and $\eta =0$ (black line). The other used parameters are: $\alpha l=400\gamma$ and $\Delta _{p}=10\gamma$.

In this section, before presenting the numerical results, it is desirable to point out some important considerations; we consider one possible realistic example to observe the phenomena using the $D_{2}$ line of sodium atoms. The ground state is specified as $|1\rangle =\left| 3 S_{1/2},F=1\right\rangle$ and the two upper states are determined as $|2\rangle =\left| 3 P_{3/2},F=0\right\rangle$ and $|3\rangle =\left| 3 P_{3/2},F=1\right\rangle$, respectively. All the used parameters are scaled by the decay rate ($\gamma _{1}=\gamma _{2}=\gamma$) and its value is equal to $\gamma =2\pi \times 9.8 MHz$. It was experimentally shown that an inverted Y-type atomic system, in two-photon resonance condition, is similar to a V-type system with SGC^34,41^. So, the same experimental setup can be basically used to observe the role of SGC in the generation of OL. We introduce three important features for achieving proper optical limiter: (i) the limiting threshold (i.e. the incident intensity when the transmission dropped down to half of the maximum value), (ii) limiting amplitude (i.e. the minimum transmission during the region of power limiting effect) and (iii) initial transmission (i.e. output intensity is proportional to incident intensity). Here, we present our results describing the transmission behavior of the system based on numerically solving the steady-state density matrix and field-propagation equations. Figure 2 shows the transmission of the probe field versus the incident intensity for the different values of the SGC parameter: $\eta =0.99\gamma$ (blue line), $\eta =0.95\gamma$ (purple line), $\eta =0.90\gamma$ (green line), $\eta =0.8\gamma$ (yellow line), $\eta =0.5\gamma$ (red line) and $\eta =0$ (black line). The other used parameters are: $\alpha l=400\gamma$ and $\Delta _{p}=10\gamma$. Note that the SGC parameter has a constant value for a quantum system in free space. However, it can be changed by considering the quantum system near the surface of the plasmonic nanostructure^26,42^.

As shown in Fig. 2, by increasing the incident intensity, the atomic system experiences the three regions for the different values of SGC parameters. In the first region, the transmission of the probe field varies linearly with respect to the incident intensity. In this situation, the slope of the transmission curve versus the incident intensity is equal to zero ($\frac{dT}{dI_{p}}=0$), leading to the proportion of output intensity with incident intensity, obeying the Beer–Lambert law. It is seen that linear treatment of the transmission varies in the specified range of the incident intensity for the different values of SGC. An investigation of Fig. 2 shows that the growth of the SGC effect leads to an increase of the initial transmission; in fact, the initial transmission can be controlled by the SGC effect. Thus, the control of the initial transmission by the SGC effect is considered as an advantage for protecting delicate optical instruments from optical damage; lower initial transmission has a crucial role in limiting performance. Notice that the prominent features; i.e., low-limiting threshold and low-limiting amplitude, low-initial transmission, and low-switching threshold can be investigated for achieving an ideal optical limiter. In the second region, the transmission of the probe field decreases by increasing the incident intensity, and the slope of the transmission gets a negative value ($\frac{dT}{dI_{p}}<0$), describing the OL region. This attenuation continues until transmission reaches its minimum, and the system experiences the maximum variation of limiting by increasing the SGC effect, leading to an efficient optical limiter. As shown in Fig. 2, the same switching and limiting thresholds can be observed for various values of the SGC effect, so the limiting amplitude can be described as OL efficiency. The corresponding plot for $\eta =0.99\gamma$ indicates better efficiency of the limiting behavior, while for weak SGC effect, transmission grows by increasing the incident intensity and the system possesses prominent SA behavior. In the third region, the transmission increases with growing the incident intensity, and the slope of the transmission gets a positive value ($\frac{dT}{dI_{p}}>0$), describing the SA domain.

**Figure 3:**
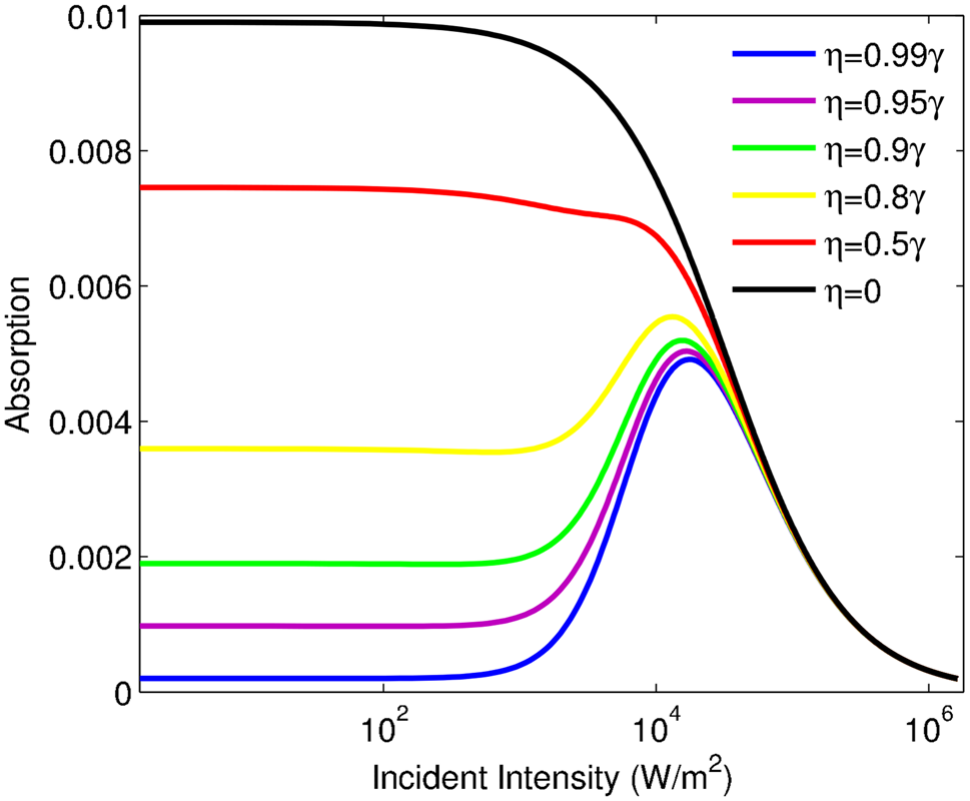
Absorption of the probe field versus the intensity of the incident field for different values of $\eta$: $\eta =0.99\gamma$ (blue line), $\eta =0.95\gamma$ (purple line), $\eta =0.90\gamma$ (green line), $\eta =0.8\gamma$ (yellow line), $\eta =0.5\gamma$ (red line) and $\eta =0$ (black line) for off-resonance condition, i.e., $\Delta _p=10\gamma$.

To fully investigate the SGC effect on the optical limiting behavior, the probe field absorption is explained. Figure 3 demonstrates the absorption of the probe field versus the intensity of the incident field for different values of $\eta$: $\eta =0.99\gamma$ (blue line), $\eta =0.95\gamma$ (purple line), $\eta =0.90\gamma$ (green line), $\eta =0.8\gamma$ (yellow line), $\eta =0.5\gamma$ (red line) and $\eta =0$ (black line). The other taken parameters are: $\alpha l=400\gamma$ and $\Delta _{p}=10\gamma$. As shown in Fig. 3, for the weak SGC effect, the SA region is induced in the desired system and the absorption is attenuated by increasing the incident intensity. While in the presence of a strong SGC effect, the absorption of the system takes an increasing trend in a certain interval of the incident intensity. Moreover, the intensity of the induced RSA region does not vary for the different values of the SGC effect. Figures 2 and 3 have their own information. In Fig. 2, we are going to study important features of the presented atomic optical limiter such as initial transmission, limiting amplitude, and OL threshold. While Fig. 3 depicts the induced RSA by applying the SGC effect between two excited energy levels. It means that an investigation of OL’s important features is not possible by considering only Fig. 3.

**Figure 4:**
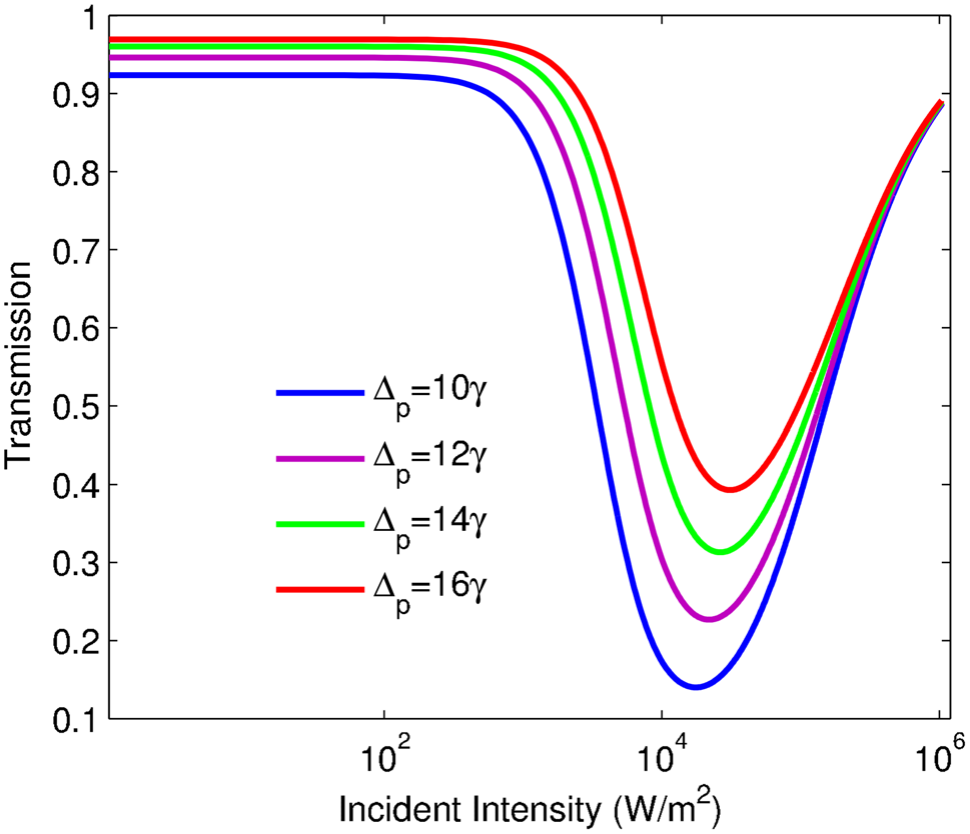
Transmission of the probe field intensity versus the incident intensity for different values of $\Delta _{p}$: $\Delta _{p}=10$ (blue line), $\Delta _{p}=12\gamma$ (purple line), $\Delta _{p}=14\gamma$ (green line) and $\Delta _{p}=16\gamma$ (red line). The other taken parameters are: $\alpha l=400\gamma$, $\eta =0.99\gamma$.

In the following, we proceed to investigate the detuning effect of the probe laser field on the OL behavior. Figure 4 illustrates the variation of the transmission function versus the incident intensity for various values of the probe field detuning $\Delta _{p}$: $\Delta _{p}=10\gamma$ (blue line), $\Delta _{p}=12\gamma$ (purple line), $\Delta _{p}=14\gamma$ (green line) and $\Delta _{p}=16\gamma$ (red line). The other taken parameters are $\alpha l=400\gamma$, $\eta =0.99\gamma$. An investigation on this figure demonstrates that the OL region is established in a wide range of the incident intensity by increasing $\Delta _{p}$. As shown in Fig. 4, the initial transmission, the OL threshold, and also the switching threshold for the RSA to SA conversion can be increased by growing $\Delta _{p}$. Notice that the prominent features of an ideal optical limiter; i.e., low-limiting threshold and low-limiting amplitude and low-switching threshold, can be more readily extracted by choosing smaller rates of $\Delta _{p}$, the corresponding plot for $\Delta _{p}=10\gamma$ indicates better efficiency of the limiting behavior.

**Figure 5:**
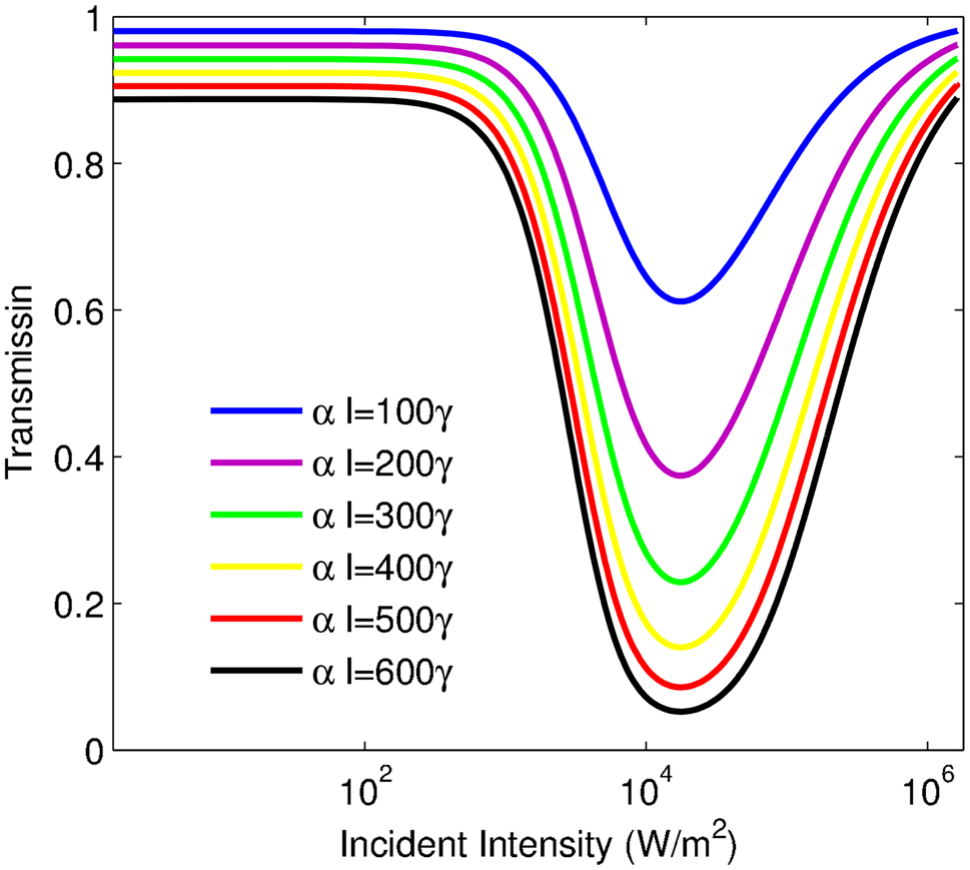
Transmission of the probe field intensity versus the incident intensity for different values of $\alpha l$ in $I_{c}=0.5850 W/m^2$. $\alpha l=100\gamma$ (blue line), $\alpha l=200\gamma$ (purple line), $\alpha l=300\gamma$ (green line), $\alpha l=400\gamma$ (yellow line), $\alpha l=500\gamma$ (red line) and $\alpha l=600\gamma$ (black line). The other used parameters are: $\eta =0.99\gamma$, and $\Delta _{p}=10\gamma$.

Now, we are going to introduce resonance absorption for achieving better efficiency of an optical limiter. The resonance absorption, $\alpha l$, depends on the atomic density and medium length, and its effect on the transmission is displayed versus the incident intensity in Fig. 5. Figure 5 indicates an increase of $\alpha l$ leads to an attenuation of the intensity of the transmission and makes an efficient limiting behavior. As proved in Fig. 5, the initial transmission and limiting threshold, and the limiting amplitude attenuate by growing the atomic density or the medium length. According to Fig. 5, an increase of $\alpha l$ has a crucial role in the improvement of the efficiency of an optical limiter. Also, the same switching thresholds can be shown due to the same trend of the resulted curves. It means that RSA to SA conversion of all curves occurs in the same incident intensity by growing $\alpha l$. In the next step, we are interested in describing the ruling physics concepts, thus the physics of phenomena can be explained by solving the density matrix equations at the steady-state. One may obtain an analytical expression for the imaginary part of $\rho _{21}$ as

11 $$\begin{aligned} \Im [\rho _{21}]=\frac{(1-\eta ^2)(4+ \Delta _p^2)\Omega _{p}+2(2-\eta ^2)\Omega _{p}^3+\Omega _{p}^5}{\Delta _p^4+\Delta _p^2\Omega _{p}^4+2\Omega _{p}^6}, \end{aligned}$$

where all parameters are scaled by $\gamma _{1}=\gamma _{2}=\gamma$. For small values of $\eta$ the first linear term in the numerator is dominant, but for higher values of $\eta$ the higher orders play a major role in establishing the reverse saturable absorption induced by the vacuum induced coherence. Moreover, Eq. (11) predicts the SA for the exact resonance condition,i.e., $\Delta _p=0$. The RSA and SA regions are determined by the minimum value of transmission which happens in $\Omega _p=\root 3 \of {\gamma \Delta _p^2}$. The analytical results are in good agreement with the numerical approach.

**Figure 6:**
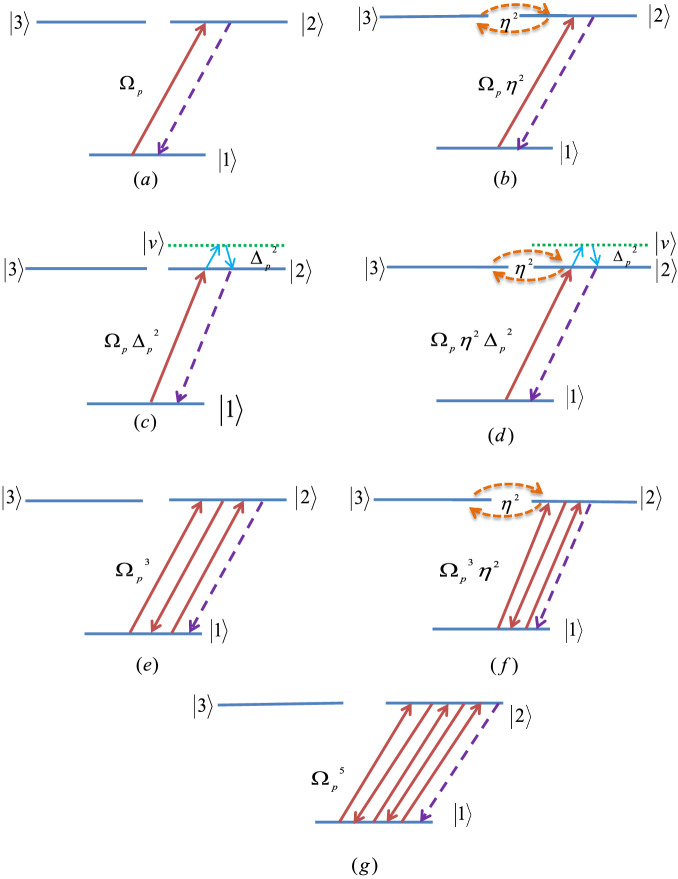
The different transition paths to generate the probe coherence in which the parts (**a–d**) is related to the linear terms. The state $\mid v\rangle$ denotes the virtual energy state. The parts (**e–g**) explain the higher order contributions. The detail of transitions is displayed in the Table 1.

To interpret the physical concept of the phenomenon, we are going to describe the transition paths related to the various terms in the numerator of the Eq. (11). Figure 6 shows the different transition paths to generate the probe coherence in which the parts (a)-(d) are related to the linear terms. The parts (e–g) explain the higher order contributions. According to Eq. (11), the SGC-induced OL is described based on the transition terms $\Omega _{p}$, $\Omega _{p}\eta ^2$, $\Omega _{p} \Delta _{p}^{2}, \Omega _{p}\Delta _{p}^2\eta ^{2}$, $\Omega _{p}^{3}$, $\Omega _{p}^{3}\eta ^2$ and $\Omega _{p}^{5}$. The first term is the contribution of the direct response of the medium to the probe field. The term $\Omega _{p}\eta ^2$ corresponds to a three-electron transition through $|1\rangle {\overset{\Omega _{p}}{\rightarrow }}|2\rangle {\overset{\eta }{\rightarrow }} |3\rangle {\overset{\eta }{\rightarrow }}|2\rangle$. The term $\Omega _{p}^{2}\Delta _{p}^2$ describes a three-electron transition through $|1\rangle {\overset{\Omega _{p}}{\rightarrow }}|2\rangle {\overset{\Delta _p}{\rightarrow }} |V\rangle {\overset{\Delta _p}{\rightarrow }}|2\rangle$, where $|V\rangle$ shows a virtual energy level. The detail of transitions related to the different terms of Eq. (11) is shown in Table 1.

**Table 1:** The detail of transitions related to Fig. 6.

Figure 6a	$\Omega _{p}$	$|1\rangle {\overset{\Omega _{p}}{\rightarrow }}|2\rangle$
Figure 6b	$\Omega _{p}\,\eta ^{2}$	$|1\rangle {\overset{\Omega _{p}}{\rightarrow }}|2\rangle {\overset{\eta }{\rightarrow }}|3\rangle {\overset{\eta }{\rightarrow }}|2\rangle$
Figure 6c	$\Omega _{p}\,\Delta _{p}^{2}$	$|1\rangle {\overset{\Omega _{p}}{\rightarrow }}|2\rangle {\overset{\Delta _{p}}{\rightarrow }}|V\rangle {\overset{\Delta _{p}}{\rightarrow }}|2\rangle$
Figure 6d	$\Omega _{p}\,\eta ^{2}\,\Delta _{p}^{2}$	$|1\rangle {\overset{\Omega _{p}}{\rightarrow }}|2\rangle {\overset{\eta }{\rightarrow }}|3\rangle {\overset{\eta }{\rightarrow }}|2\rangle {\overset{\Delta _{p}}{\rightarrow }}|V\rangle {\overset{\Delta _{p}}{\rightarrow }}|2\rangle$
Figure 6e	$\Omega _{p}^{3}$	$|1\rangle {\overset{\Omega _{p}}{\rightarrow }}|2\rangle {\overset{\Omega _{p}^{*}}{\rightarrow }}|1\rangle {\overset{\Omega _{p}}{\rightarrow }}|2\rangle$
Figure 6f	$\Omega _{p}^{3}\,\eta ^{2}$	$|1\rangle {\overset{\Omega _{p}}{\rightarrow }}|2\rangle {\overset{\Omega _{p}^{*}}{\rightarrow }}|1\rangle {\overset{\Omega _{p}}{\rightarrow }}|2\rangle {\overset{\eta }{\rightarrow }}|3\rangle {\overset{\eta }{\rightarrow }}|2\rangle$
Figure 6g	$\Omega _{p}^{5}$	$|1\rangle {\overset{\Omega _{p}}{\rightarrow }}|2\rangle {\overset{\Omega _{p}^{*}}{\rightarrow }}|1\rangle {\overset{\Omega _{p}}{\rightarrow }}|2\rangle {\overset{\Omega _{p}^{*}}{\rightarrow }}|1\rangle {\overset{\Omega _{p}}{\rightarrow }}|2\rangle$

**Figure 7:**
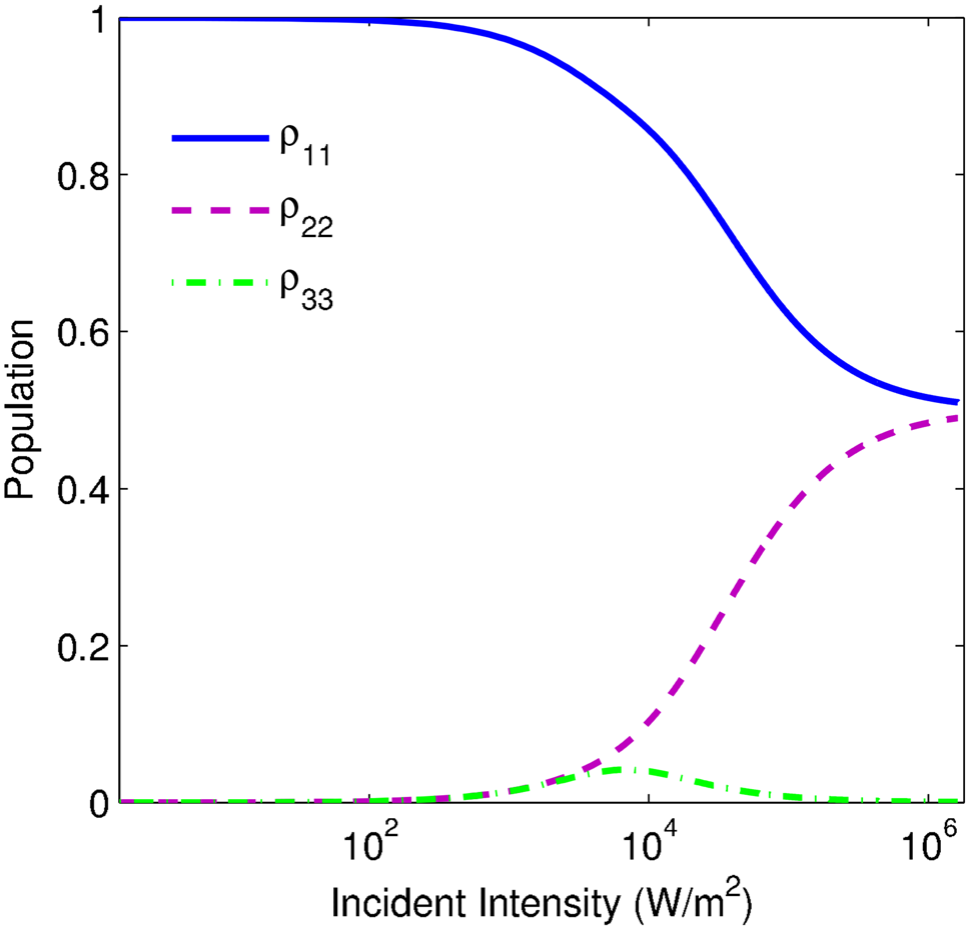
The population distribution of energy levels versus the incident intensity for $\eta =0.99\gamma$. The other taken parameters are the same as Fig. 3.

Here, in order to infer the physical concepts for OL behavior, we proceed to investigate the population distribution. The taken parameters are the same as Figs. 3 and 7 demonstrates that the population transfer does not occur in the energy levels for the case of low incident intensities $I_{p}<10^3 {\text {W/m}}^2$. While by increasing the incident intensity ($I_{p}>10^3 {\text {W/m}}^2$), the population in the $|1\rangle$ is drastically decreased and is accumulated in the energy levels $|2\rangle$ and $|3\rangle$. It means that state $|3\rangle$ starts to deplete the accumulated population in levels $|2\rangle$. In general, this transfer population between the energy levels continues until the same population distribution is observed in $|1\rangle$ and $|2\rangle$.

**Figure 8:**
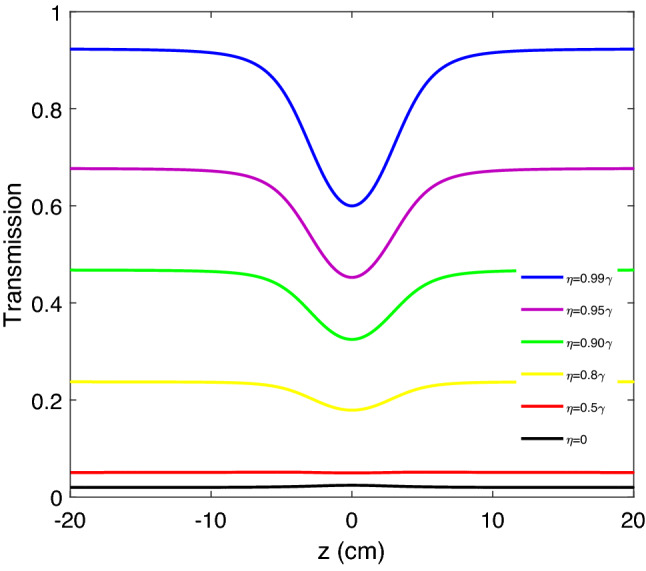
Z-scan measurement of induced OL in the presence of the SGC effect for certain values of the probe incident intensity $I_{0}=6.656\times 10^3 {\text { W/m}}^2$. The wavelength of the Gaussian probe field is presented at 589.158 nm for different values of the SGC effect.

Now, we are interested in introducing a Z-scan technique to analyze the features of optical limiting behavior. The open aperture Z-scan configuration is formed from a laser, thin lens, diaphragm, and detector. In this configuration, a Gaussian laser beam propagating in the z-direction can be focused by using the lens. Notice that transmission is considered as a function of the *z* position. So, when the sample is moved along the z-direction ($- z$ to $+ z$), it experiences the maximum intensity of the laser beam in the focal point ($z=0$). By getting away from the focal point in either direction, the beam intensity remarkably decreases. The distribution of intensity in the sample leads to varying the refractive index. It is investigated that in long-distance of a focal point, the variation of refractive index is minimum, thus the transmission can vary as linearly. But when the sample is moved forward to the focal point, the variation of the refractive index versus the intensity distribution increases and this variation leads to increasing (decreasing) the output intensity. Based on this description, the Z-scan shows peaks (dips) in the focal point. The observed peaks (dips) around the focal point define the RSA (SA) region. Out of the focal point, the transmission has linear behavior and gets the constant value. But for the other values of the used parameters, we can observe some minimums (maximums) on the wings. The lateral minima are seen around the central peak of the SA means the transformation from the RSA to SA in minimum. The intensity of the Gaussian laser beam is applied in the Z-scan technique given by:

12 $$\begin{aligned} I_{p}(z, r)=I_{0} \frac{w_{0}^{2}}{w^{2}(z)} \exp \left[ -\frac{2 r^{2}}{w^{2}(z)}\right], \end{aligned}$$

where $r=\sqrt{x^2+y^2}$ is the distance of the point position from the axis of the beam. In Eq. (12), $I_{0}$ is described as the amplitude of input probe beam. The beam radius is represented as $w(z)=w_{0}\left[ 1+\left( z / z_{0}\right) ^{2}\right] ^{1/2}$. Also, $w_0$ and $z_{0}=\pi w_{0}^{2} / \lambda$ determine the beam waist and the rate of the diffraction length for the Gaussian probe beam, respectively. The beam radius in the focal point is described by $w_{0}$. Figure 8 shows the validity of our obtained results in $I_{p_{0}}=6.656\times 10^3{\text{ W/m}}^2$ for different values of the SGC. The wavelength of the Gaussian probe field in the presented Z-scan measurement is 589.158 nm, and the width of the Gaussian probe field is considered 0.1 mm. The other used parameters are $\alpha l=400\gamma$ and $\Delta _{p}=10\gamma$. Notice that the intensity of the focal point is selected based on the strength of the SGC effect. As shown in Fig. 8, an increase of the SGC effect leads to the observation of the dip in the focal point while choosing the weak SGC ($\eta <0.5\gamma$), there is no dip in the focal point. It is noticeable that increasing the SGC effect plays a crucial role in inducing OL behavior.

## Conclusion

We investigated the RSA and OL behavior in a three-level V-type quantum system considering the SGC effect. It was shown that when the SGC effect is ignored, the SA is the dominant phenomenon in the system. By applying the SGC effect, it is demonstrated that the SA switches to the RSA. In the RSA region, we showed that the transmission of the probe field attenuates dramatically by increasing the SGC effect, which leads the optical limiter more efficient. Moreover, it is illustrated that the OL threshold and OL region as the major characteristics of the optical limiter can be controlled by the SGC effect. It was also shown that the applied field properties such as detuning can modify the optical limiter efficiency. The analytical results determined that the Kerr nonlinearity generated by the SGC effect is the main mechanism in the system evolution. Moreover, it is shown that the SA is dominant in one-photon resonance condition. In addition, it was depicted that an increase in the atomic density and length of the medium makes the optical limiter more efficient. Ultimately, the theoretical Z-scan experiment was presented to confirm the validity of the obtained results. The proposed scheme can be used to design controllable atomic optical limiters for optical devices with different sensitivities.

## Data Availability

The datasets used and/or analysed during the current study are available from the corresponding author on reasonable request.
